# Concordance and test-retest consistency of sleep biomarker-based neurodegenerative disorder profiling

**DOI:** 10.1038/s41598-024-82528-y

**Published:** 2024-12-28

**Authors:** Daniel J. Levendowski, Debby Tsuang, Lana M. Chahine, Christine M. Walsh, Chris Berka, Joyce K. Lee-Iannotti, David Salat, Corrine Fischer, Gandis Mazeika, Bradley F. Boeve, Luigi Ferini Strambi, Simon J. G. Lewis, Thomas C. Neylan, Erik K. St. Louis

**Affiliations:** 1 Advanced Brain Monitoring, 2237 Faraday Avenue, Suite 100, Carlsbad, CA 92008 USA; 2 VA Puget Sound, Seattle, WA USA; 3 University of Pittsburgh, Pittsburgh, PA USA; 4 University of California, San Francisco, CA USA; 5 Banner University Medical Center Phoenix, Phoenix, AZ USA; 6 Massachusetts General Hospital, Charlestown, MA USA; 7 St. Michael’s General Hospital, Toronto, Canada; 8 Mayo Clinic College of Medicine and Science, Rochester, MN USA; 9 Universitá Vita-Salute San Raffaele, Milano, Italy; 10 Macquarie University, Sydney, Australia

**Keywords:** Neurodegenerative disease, Sleep biomarkers, Alzheimer’s disease, Parkinsonian spectrum disorders, REM sleep behavior disorder, Non-REM hypertonia, Diagnostic markers, Neurological disorders

## Abstract

Biomarkers that aid in early detection of neurodegeneration are needed to enable early symptomatic treatment and enable identification of people who may benefit from neuroprotective interventions. Increasing evidence suggests that sleep biomarkers may be useful, given the bi-directional relationship between sleep and neurodegeneration and the prominence of sleep disturbances and altered sleep architectural characteristics in several neurodegenerative disorders. This study aimed to demonstrate that sleep can accurately characterize specific neurodegenerative disorders (NDD). A four-class machine-learning algorithm was trained using age and nine sleep biomarkers from patients with clinically-diagnosed manifest and prodromal NDDs, including Alzheimer’s disease dementia (AD = 27), Lewy body dementia (LBD = 18), and isolated REM sleep behavior disorder (iRBD = 15), as well as a control group (CG = 58). The algorithm was validated in a total of 381 recordings, which included the training data set plus an additional AD = 10, iRBD = 18, Parkinson disease without dementia (PD = 29), mild cognitive impairment (MCI = 78) and CG = 128. Test–retest consistency was then assessed in LBD = 10, AD = 9, and CG = 46. The agreement between the NDD profiles and their respective clinical diagnoses exceeded 75% for the AD, LBD, and CG, and improved when NDD participants classified Likely Normal with NDD indications consistent with their clinical diagnosis were considered. Profiles for iRBD, PD and MCI participants were consistent with the heterogeneity of disease severities, with the majority of overt disagreements explained by normal sleep characterization in 27% of iRBD, 21% of PD, and 26% of MCI participants. For test–retest assignments, the same or similar NDD profiles were obtained for 88% of LBD, 86% in AD, and 98% of CG participants. The potential utility for NDD subtyping based on sleep biomarkers demonstrates promise and requires further prospective development and validation in larger NDD cohorts.

## Introduction

Insufficient and poor-quality sleep negatively impacts cognitive processing and memory consolidation and likely contributes to neurodegeneration as a result of increased sleep deprivation-related beta amyloid and tau production during extended wakefulness, and/or decreased clearance during sleep reduction^1–3^. The accumulation of pathogenic proteins underlying neurodegeneration can begin in midlife, 15- to 20-years prior to the manifestation of prodromal or early neurodegenerative disorder (NDD) symptoms and signs^4^. The lack of deep sleep and possibly sleeping in the supine position further compromises nightly glymphatic clearance of neurotoxic proteins from the brain^5,6^.

Increasing evidence suggests that sleep has a bi-directional relationship with NDD^4,7^. Isolated REM sleep behavior disorder (iRBD), an established prodromal synucleinopathy in most older adults, is characterized by complex vocal and motor behaviors during rapid eye movement (REM) sleep and confirmed by the presence of REM sleep without atonia (RSWA)^8,9^. Electroencephalography (EEG) background slowing including excessive theta and delta frequency activity is present in Alzheimer’s disease (AD) and Parkinson’s disease (PD) patients with cognitive decline^10,11^. Reduced REM sleep duration has been linked to compromised memory consolidation and observed in both AD and Lewy body dementia (LBD) patients^11–13^. Non-REM sleep spindle activity influences memory consolidation and decreased sleep spindle activity has been associated with increased tau in cognitively normal elderly adults^14,15^. Additional sleep-related measures, including autonomic characteristics and non-REM hypertonia, have been described in Parkinsonian spectrum disorders^16–18^.

Previously, our research consortium demonstrated that combined NREM sleep biomarkers (sleep spindles and non-REM hypertonia) could distinguish several common NDD subtypes including AD, LBD, PD, iRBD, mild cognitive impairment (MCI), and progressive supranuclear palsy from cognitively-normal controls^13^. In the current study, we aimed to examine the accuracy and longitudinal consistency of NDD profiling based on probabilities across four diagnostic categories using a machine-learning classifer trained with an expanded number of sleep biomarkers.

## Methods

### Participants

The detailed criteria used to diagnose the NDD cohorts and to characterize participants in the CG were previously described (Table 1)^6,13,17,18^. Patients were diagnosed with AD according to DSM-5 criteria and/or McKhann et al. criteria^19^. The LBD group included those diagnosed with dementia with Lewy bodies (DLB; *n* = 14) according to the McKeith criteria^20^ or Parkinson’s disease dementia (*n* = 4). The PD participants met either the most recent Movement Disorder Society criteria or United Kingdom PD Society Brain Bank clinical diagnostic criteria^21^. Those with MCI had subjective complaints and objective cognitive impairment with a Mini-Mental State Examination (MMSE) score between 24 and 27. Participants in the pSYN training data set al.l were diagnosed with iRBD by PSG, however, a portion of the pSYN cohort in the validation group were suspected but unconfirmed RBD. The CG used for model development and biomarker receiver operating characteristic (ROC-AUC) assessments had an MMSE score *≥* 28. For the CG validation cohort, 109 had an MMSE *≥* 28 with the balance having subjective complaints of poor sleep quality but no memory or cognitive complaints with 67 of the 77 subsequently diagnosed with an apnea/hypopnea index *≥* 5 events/h. All authors contributed data to this study with all research performed in accordance with the Declaration of Helsinki. Prospectively acquired studies were approved by institutional review boards at Mayo Clinic VA Puget Sound, University of Pittsburgh, Banner University Medical Center, St. Michaels Hospital, Massachusetts General Hospital, University of California San Francisco, and Advanced Brain Monitoring while the need to obtain informed consent was waived by Alpha IRB for those recordings which were retrospective in nature. Recordings used in this study were acquired between January 2017 and August 2024.

Subsets of participants from the validation cohort were longitudinally evaluated. From the CG cohort, 46 were retested in a range between 8- and 58-months. Ten LBD participants were evaluated longitudinally, with seven retests at 6-months and nine retests at 12-months. Nine AD participants completed six retests at six-months and eight retests after 12-months.

**Table 1:** Description of Group Data Used for Model Development, Biomarker Assessment, and validation of NDD profiles.

Cohort name	Label	Model development (*n*)		Validation cohort descriptors		
		Training	ROC-AUC	Size (*n*)	Age	Female
Control group	CG	58	61	186	62 *±* 9.5	46%
Alzheimers disease dementia	AD	27	29	37	73 *±* 7.8	27%
Lewy body dementia	LBD	18	18	19	70 *±* 6.2	11%
REM sleep behavior disorder	iRBD/pSYN	15	19	33	66 *±* 10.0	27%
Parkinson’s disease	PD	---	17	29	65 *±* 9.6	24%
Mild cognitive impairment	MCI	---	40	86	70 *±* 9.9	45%

### Sleep biomarkers

Recordings used to extract the sleep biomarkers were acquired with the Sleep Profiler™ (Advanced Brain Monitoring, Inc.). The device recorded frontopolar EEG from Af7-Af8, and left and right electrooculography signals from Af7-Fpz and Af8-Fpz. Pulse rate was extracted from a photoplethysmography signal obtained with reflectance-based infrared emitter/photodiode, while head movement and head position were derived from a three-dimensional accelerometer, and quantitative snoring was measured with an acoustic microphone^21^. All participants were studied in their home, with the exception of 18 iRBD participants who were studied during simultaneous in-lab PSG.

Differentiation between wake, rapid eye movement (REM) sleep, and the non-REM sleep stages N1, N2 and N3 was made using a combination of machine-learning algorithms and empirically determined rule-based thresholds applied to power spectral changes within and across 30-second epochs^22^. Additional features that helped to accurately stage sleep include automated detection of cortical and micro arousals, sleep spindles, snoring, head movements, rapid and slow rolling eye movements, and changes in EMG power extracted from the EEG that occurs during sleep onset and awakenings^13,22,23^.

The night-to-night reliability and/or capability to differentiate between controls and NDD subgroups using sleep biomarker cutoffs has been previously reported^13,17,22^. Table 2 presents the receiver operating characteristic areas under the curve (ROC-AUC), sensitivities and specificities for the sleep biomarkers based on cutoffs applied to the model development “ROC-AUC” data set (Table 2). Three biomarkers were based on conventional sleep metrics, i.e., REM sleep time, sleep efficiency and supine sleep time. The automated detection of short duration bursts in sigma and alpha power which denote sleep spindles were previously described^13^. The auto-detection of non-REM hypertonia (NRH) was based on a detailed set of algorithms that recognizes abnormally elevated electromyographic (EMG) power relative to delta, theta, and sigma bands across a minimum of four contiguous 30-second epochs^17^. The differentiation between conventional stage N3 and atypical N3 (i.e., an EEG pattern associated with delirium commonly referred to as sepsis-associated encephalopathy constituted by polymorphic delta activity) was achieved using a machine learning algorithm developed to differentiate EEG frequency ratios during N3 using delta/theta and alpha/sigma ratios^24^. Pulse rate excursions *≥* 6 beats-per-minute were used to quantify autonomic activation, with abnormally low values found to be previously associated with synucleinopathy-related autonomic dysfunction and extremely high values typically relating to sleep disordered breathing arousals^16^.

**Table 2:** Diagnostic characteristics of the individual sleep biomarkers used for NDD profiling.

Sleep Biomarker	Disease Category(s)	Unaffected Category(s)	Abnormal Cutoff	ROC-AUC	Sensitivity	Specificity
Lewy Body Dementia						
REM time	LBD	AD, PD, MCI, iRBD, CG	≤ 30 min	0.78	0.63	0.93
Spindle duration			≤ 1 min	0.74	0.84	0.63
Atypical N3			≥ 4% sleep time	0.66	0.42	0.89
Synucleinopathy						
Non-REM hypertonia	LBD, PD, iRBD	AD, MCI, CG	≥ 5% sleep time	0.74	0.70	0.79
Autonomic activation			< 10 events/h	0.65	0.54	0.75
Dementias & cognitive Impairment						
NREM relative theta	LBD, AD, MCI	PD, iRBD, CG	≥ 18.5%	0.71	0.68	0.73
NREM theta/alpha			≥ 1.40	0.63	0.52	0.74
NDD Condition						
Sleep efficiency	LBD, AD, MCI, PD, iRBD	CG	≤ 80%	0.65	0.41	0.89
Supine sleep			≥ 120 min	0.61	0.63	0.59

### Profiling NDD

The AD, LBD, iRBD and CG model development “training” data (Table 1) were submitted to a machine-learning classifier using the nine sleep biomarkers listed in Table 2 plus age. Outputs from the discriminant function analysis included partial probabilities for each of the four classes which were categorically stratified into profile labels using the rules described in Table 3. Given that REM sleep without atonia is not a variable used for NDD profiling in this study, the label prodromal synucleinopathy (pSYN) was assigned to partial probabilities for the class trained using iRBD participants.

**Table 3:** Assignment rules for NDD profiles based on 4-Class probability outputs.

Group	Rules for NDD Profile Assignments		
Probable			
Normal	CG ≥ 0.70	CG ≥ 0.65, LBD,AD, pSYN all ≤ 0.25	
AD	AD ≥ 0.70	AD ≥ 0.50, LBD, CG, pSYN all ≤ 0.20	
LBD	LBD ≥ 0.70	LBD ≥ 0.50, pSYN ≥ 0.15, CG & AD ≤ 0.20	
pSYN	pSYN ≥ 0.65 & AD < 0.35	pSYN ≥ 0.50, 0.20 ≥ LBD < 0.40, CG & AD < 0.20	
Mixed	AD + RBD or LBD + RBD ≥ 0.70	CG < 0.20, AD,LBD, pSYN all ≥ 0.05	
Likely			
Normal	CG ≥ 0.50, AD, LBD, pSYN all < 0.30	0.35 ≥ CG < 0.70, AD, LBD, pSYN all ≤ 0.25	
AD	AD ≥ 0.50, CG < .40	AD ≥ 0.55, CG < 0.45	
LBD	LBD ≥ 0.45, CG < 0.40	(LBD + pSYN-CG) ≥ 0.15	
pSYN	pSYN ≥ 0.45, CG < 0.40	pSYN ≥ 0.55, CG < 0.45	(pSYN + LBD-CG) ≥ 0.15
Mixed	CG ≤ 40, (AD + RBD-CG) ≥ 0.20	CG ≤ 40, (AD + LBD-CG) ≥ 0.20	CG < 40, (LBD + pSYN-CG) ≥ 0.20
Likely Normal with Indications of			
AD	CG ≥ 0.40, AD < 0.60, LBD, pSYN < 0.05	CG ≥ 0.45, 0.25 ≥ AD < 0.50, LBD, pSYN < 0.20	
LBD	CG > 0.40, LBD < 0.60, AD, pSYN < 0.05	CG > 0.45, 0.25 > LBD < 0.50, AD, pSYN < 0.20	
pSYN	CG > 0.40, pSYN < 0.60, LBD, pSYN < 0.05	CG > 0.45, 0.20 ≥ LBD < 0.50, AD, pSYN < 0.20	
Mixed	≥ 0.35 > CG < 0.45,(LBD + AD-CG) ≥ 0.10	AD + LBD, AD + pSYN, LBD + pSYN all ≥ 0.30	

**Fig. 1:**
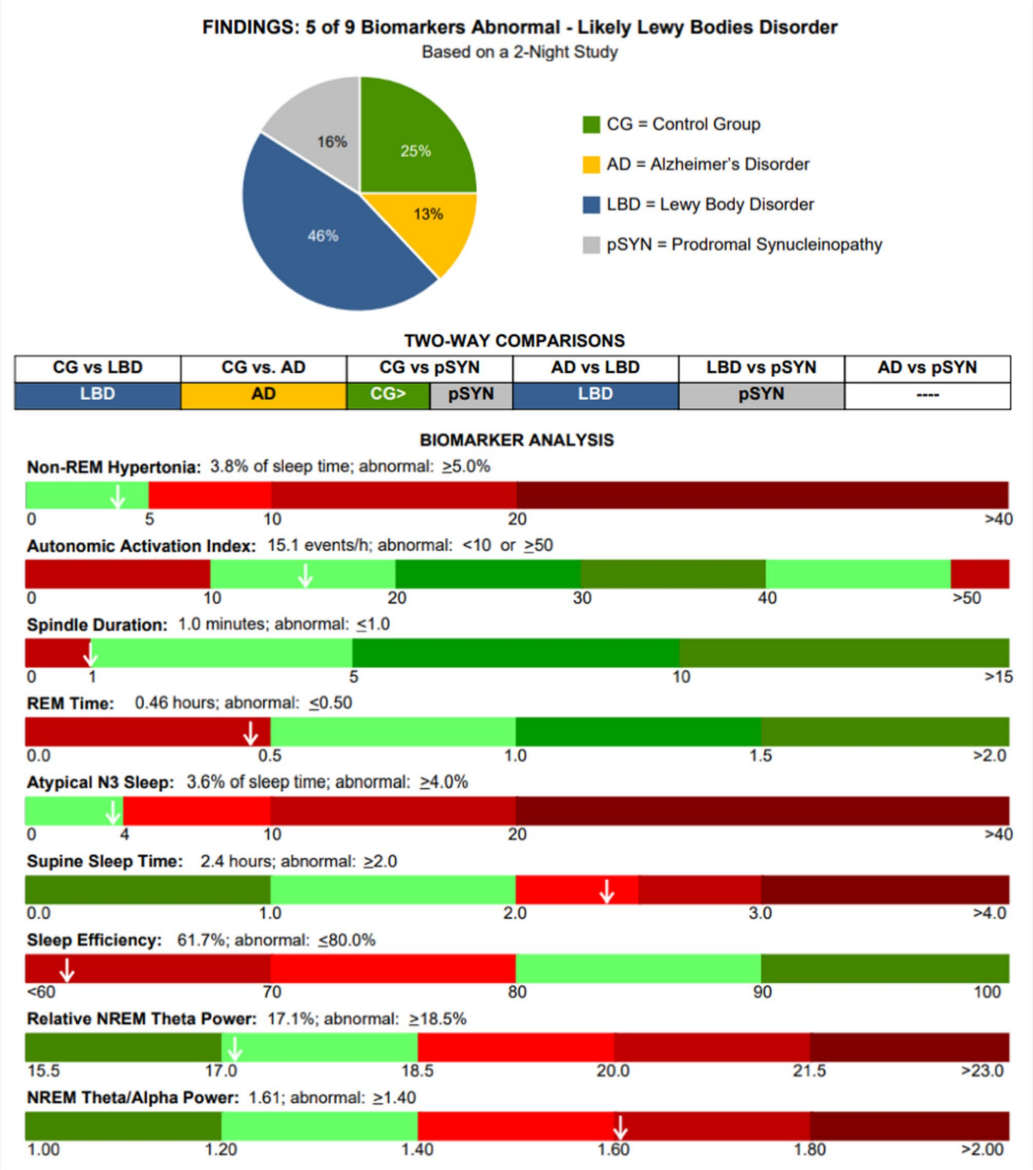
Sample report for a PD patient classified as Probable Mixed with seven abnormal biomarkers. Based on the two-class models, LBD was superior to AD, pSYN was superior to LBD and CG and pSYN were approximately equivalent.

The biomarker values submitted for calculation in the 4-class model were weight-averaged when multiple nights were acquired (e.g., Biomarker value Night 1 x (sleep times Night 1 / Nights 1 + 2) + (biomarker value Night 2 x (sleep times Night 2 / Nights 1 + 2) etc.). Additional criteria were applied to the classification model in order to reduce the likelihood of a misclassification when applied to data the model was not trained to recognize. For example, given the youngest age within the CG training data set was 55 years, all NDD profiles for those younger than 55 years were calculated using 55 years. The autonomic activation index (AAI) was only included when the percentage of technically adequate pulse detections during epochs staged as non-REM or REM was *≥* 65% and the maximum AAI submitted for the calculation was 55 events/h.

Six two-class models were developed for each permutative combination (e.g., CG vs. AD, CG vs. LBD, etc.) to assist in future interpretation of probability edge effects and/or an assignment of a “Mixed” classification. Each two-class output was assigned a winner based on the probability, and when both probabilities were between 40 and 60%, duel winners were assigned. Figure 1 presents a sample report with the overall classification and probability distributions followed by presentation of the two-class winners (with CG and pSYN as dual winners), and finally, classification results are provided for the individual sleep biomarkers, with abnormal cutoffs based on those shown in Table 2.

NDD profiles were tallied according to diagnostic agreement (e.g., classified Probably or Likely Normal for the CG, Probable or Likely NDD, etc.). Overt disagreements included CG classified with a Probable NDD, and patients classified as Probably Normal or assigned an NDD subtype inconsistent with the diagnosis e.g., diagnosed AD but classified as LBD, etc. Trending toward disagreement included CG classified with a likely NDD. Trended-toward-agreement included NDD groups classified as Likely Normal with indications consistent with their clinical diagnosis.

## Results

Distributions of sleep biomarker characteristics of the validation data set used for the NDD profiles are presented in Table 4.

**Table 4:** Distributions of sleep biomarkers (mean *±* SD) for the validation data set (*n* = 381).

Sleep Biomarker	CG	AD	LBD	pSYN	PD	MCI
REM time, min	87.9 *±* 29.3	65.4 *±* 29.7	30.4 *±* 33.9	60.4 *±* 43.5	55.8 *±* 32.6	75.8 *±* 35.4
Spindle duration, min	7.7 *±* 11.8	3.3 *±* 5.8	2.8 *±* 9.0	3.6 *±* 4.6	4.1 *±* 5.6	5.7 *±* 9.0
Atypical N3, %	1.2 *±* 2.3	2.2 *±* 3.7	9.1 *±* 12.1	2.0 *±* 3.9	3.7 *±* 8.5	1.8 *±* 3.0
Non-REM hypertonia, %	3.9 *±* 5.7	2.9 *±* 4.5	16.6 *±* 13.1	13.3 *±* 12.6	10.6 *±* 9.7	4.2 *±* 5.4
Autonomic activation index, events/h	26.1 *±* 20.1	22.1 *±* 17.8	11.5 *±* 11.0	16.9 *±* 13.9	12.5 *±* 13.6	22.5 *±* 17.5
NREM relative theta, %	17.7 *±* 1.5	19.7 *±* 2.1	19.8 *±* 1.7	17.3 *±* 1.4	18.7 *±* 2.0	18.7 *±* 2.1
NREM theta/alpha, ratio	1.26 *±* 0.24	1.48 *±* 0.37	1.74 *±* 0.39	1.19 *±* 0.28	1.41 *±* 0.36	1.32 *±* 0.25
Sleep efficiency, %	85.6 *±* 7.3	78.4 *±* 9.2	72.4 *±* 17.8	73.1 *±* 15.8	76.8 *±* 12.8	78.8 *±* 12.6
Supine time, min	126 *±* 130	188 *±* 111	178 *±* 146	130 *±* 98	164 *±* 88	175 *±* 116

**Fig. 2:**
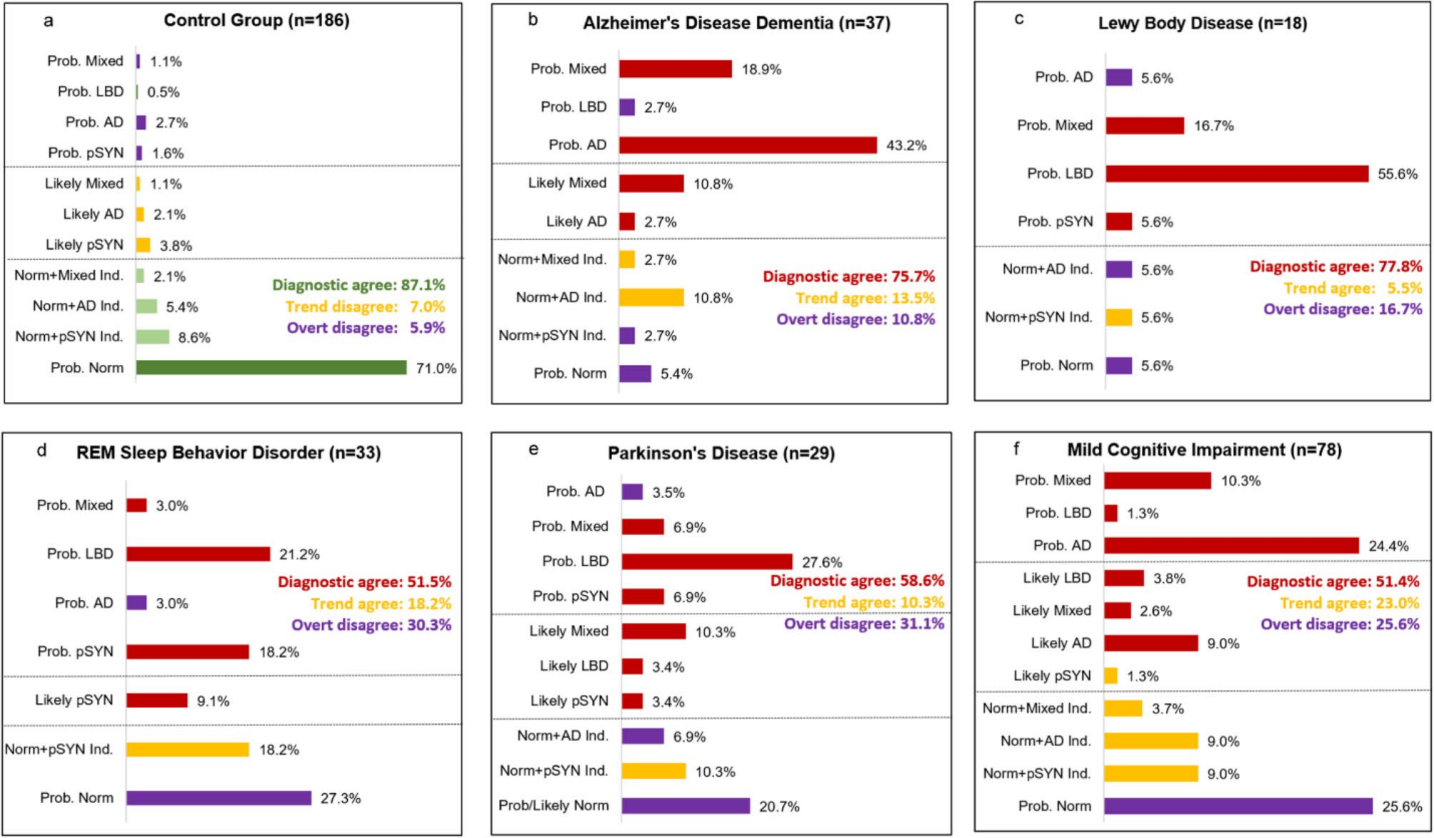
Distributions of NDD risk classifications that are color coded and tallied based on diagnostic agreement, overt disagreement, and trending toward agreement (i.e., NDD groups classified as Likely Normal with indications consistent with the clinical diagnosis) for: (**a**) control group, (**b**) Alzheimer’s disease dementia, (**c**) Lewy Body dementia, (**d**) REM sleep behavior disorder, (**e**) Parkinson’s disease, and (**f**) mild cognitive impairment. NDD groups classified as Likely Normal with indications consistent with their clinical diagnosis were considered borderline cases and classified as trended-toward-disagreement.

Figure 2 presents the distribution of profiles by group. In the CG, 87% were classified as Probably Normal or Likely Normal, and 6% were classified as having a Probable NDD (Fig. 2a). Of the AD group, 76% were assigned a Likely or Probable NDD profile (Fig. 2b). Overt disagreements included two AD participants diagnosed as Probably Normal, one Likely Normal with pSYN indication, and an AD patient characterized as Probable LBD. In the LBD group the classifications were consistent with the diagnosis in 78% of the cases, with one LBD patient characterized as Probably Normal, one Likely Normal with indications of AD, and a third case assigned Probable AD (Fig. 2c).

Classification patterns in the iRBD, PD and MCI cohorts were more heterogenous. In the iRBD cohort, 52% were characterized with a Probable or Likely NDD, while 30% were presumed to have overt disagreements based on nine classified as Probably Normal and one case classified as Probable AD (Fig. 2d). Similarly, 59% of the PD group had assignments consistent with a synucleinopathy, while 31% were classified with overt disagreements i.e., six were classified as Probably/Likely Normal, two as Likely Normal with AD indications, and one as Probable AD (Fig. 2e). For the MCI group, diagnostic agreements were obtained in 51% of the cohort, with presumed overt disagreements in 26% based on 20 MCI participants being classified as Probably Normal (Fig. 2e).

### Test-retest reliability

74% of the CG had the same NDD profile assigned at baseline and at retest (bolded in Table 5). The greatest classification shift occurred in a 44-year-old participant classified as Probably Normal at baseline and Likely pSYN four years later. One participant shifted from Likely pSYN to Likely Normal with pSYN indications (based on pSYN probabilities of 63% and 40%, respectively).

The model development CG used to train the machine learning algorithm included three participants who were excluded from the test-retest analyses due to subsequent development of decreased MMSE scores. Two subjects with MMSE scores of 30 were classified as Likely Normal with AD indications at baseline but assigned Probable AD at retest with MMSE scores of 25 and 27 at one-year and four-years, respectively. An 84-year-old participant with an MMSE score of 30 was classified as Probable AD at baseline with a probability of 0.96 and retested two years later with an AD probability of 0.95 and an MMSE of 27.

**Table 5:** Test-retest confusion matrix for CG with same NDD profiles bolded.

NDD Profiles	Prob. Norm	Norm + pSYN Ind	Norm + AD Ind.	Norm + Mixed Ind.	Likely pSYN	Likely AD	Prob. AD	Prob. Mixed
Retest	Test (*n* = 46)							
Prob. Norm	**59% (27)**	4.5% (2)		2% (1)				
Norm + pSYN Ind.	7% (3)	**7% (3)**			2% (1)			
Norm + AD Ind.	4.5% (2)		**2% (1)**	2% (1)				
Likely pSYN	2% (1)				**2% (1)**			
Likely Mixed						**2% (1)**		
Prob.AD							**2% (1)**	
Prob. LBD								2% (1)

Seventy-percent of the LBD patients were assigned the same NDD profile when retested (Bolded in Table 6). One LBD patient was characterized as Probably Normal at baseline and again at six-month retest. One patient slightly improved from Probable Mixed to Likely Mixed. A third LBD patient shifted twice, from Probable AD to Probable Mixed at six-months, and to Probable LBD at 12-months.

For the AD participants, 79% were assigned the same NDD profile upon retest (bolded in Table 6). One patient assigned Likely Normal with AD indications at baseline shifted to Probable Mixed after six-months. One patient contributed two misclassifications based on improvements from Probable AD at baseline, to Likely AD at six-months, and Likely Normal with AD indications at 12-months. One patient diagnosed with AD was characterized twice as Probably Normal, at baseline and again 12-months later.

**Table 6:** Test-retest confusion matrix for LBD and AD with same NDD profiles bolded.

NDD Profiles	Prob. Norm	Norm + AD Ind	Prob. LBD	Prob. Mixed	Prob. AD
LBD Retest	LBD Test (*n* = 17)				
Prob. Norm	**6% (1)**				
Likely Mixed				6% (1)	
Prob. LBD			**41% (7)**		6% (1)
Prob. Mixed			12% (2)	**23% (4)**	6% (1)
AD Retest	AD Test (*n* = 14)				
Prob. Norm.	**7% (1)**				
Norm + AD Ind.					7% (1)
Likely AD					7% (1)
Prob. AD					**65% (9)**
Prob. Mixed		7% (1)		**7% (1)**	

## Discussion

To our knowledge, this is the first study to develop and validate combined NREM and REM sleep biomarkers for NDD subtyping. The agreement between the NDD profiles and respective clinical diagnosis categories exceeded 75% for AD, LBD, and controls, and improved when classifications included borderline cases that trended toward agreement. In the iRBD, PD and MCI groups, NDD profiles were less uniform with greater disbursement toward varying phenotypic classifications. This could be consistent with a more heterogenous range of sleep disturbance and cortically-derived sleep biomarkers in prodromal AD and synucleinopathy groups (i.e., iRBD, MCI) and in PD patients. Over 20% were characterized as having normal sleep and over half were assigned profiles consistent with cognitive decline.

Factors that influence NDD profiles are beginning to emerge as a result of the longitudinal assessment of NDD sleep biomarkers. Because AD and LBD share low spindle duration and increased NREM slowing (measured by relative theta, theta/alpha and atypical N3) as conditional abnormalities, the probabilities can lack sufficient distinction, resulting in a “Mixed” classification or a potential misclassification. In some cases, the Probable AD vs. LBD assignment was made only after the severity of biomarker abnormality and/or biomarkers abnormality unique to the proteinopathies emerged.

The pSYN category was included in the model with the goal of being combined with the LBD probability to help distinguish the possible timeline for dementia phenoconversion in participants with synucleinopathies, including those possible prodromal forms of synucleinopathy having iRBD and MCI. The pSYN classification was trained using data from those diagnosed with iRBD without confirmatory RSWA that could be obtained with additional analysis of chin EMG signals from the Sleep Profiler. In one case, we observed an LBD patient classified as Probable AD, but with a high RSWA density score. Future plans for improvement of the classification model include automated detection of RSWA which can likely improve diagnostic accuracy.

The proposed classification model includes layers of information to assist with interpretation of possible NDD. The probabilities derived from the 4-class model could be reviewed in combination with the 2-class profiles to identify cases with NDD profiles very close to the edge of a different classification. The sleep biomarkers that contributed to NDD profile should also be reviewed. For example, a high autonomic activation index, abnormal spindle duration and extended supine sleep duration may suggest the need to rule out untreated OSA. The number of abnormal sleep biomarkers and the degree of specific biomarker abnormalities that contribute to an assignment may also assist in assessing possible NDD severity. Normal with Indications of a pSYN based on highly abnormal NRH could prompt a query regarding possible dream enactment behavior symptoms, and/or an assessment for RBD/RSWA.

Sleep biomarkers that contribute to an abnormal NDD profile may be impacted by medications. In our previous study of similar NDD cohorts, the use of selective serotonin and noradrenaline reuptake inhibitors were associated with increased NRH, and are known suppressors of REM sleep^13,25^. Benzodiazepines can distort sleep efficiency by increasing sleep duration^26^ and may induce pseudo-spindle activity that could confound the expected pattern of suppressed spindle activity in LBD and AD^13^.

The use of clinical diagnoses alone, without pathological confirmation or additional deeper patient phenotyping by use of functional neuroimaging or fluid biomarkers to establish NDD diagnoses is an important limitation of this study. However, the participants analyzed in this study are broadly representative of patients who are seen in clinical settings. Participants were presumed to be exclusively classifiable to one of the LBD and AD groups, despite the potential for mixed LBD and AD pathologies that would require additional confirmation by imaging, fluid biomarkers or neuropathology^27^. Use of the same LBD records for model development and validation was another limitation, due to the limited sample size in this category. While this most certainly led to inflated performance in the LBD subgroup, it should be noted that three of the sleep biomarkers used in the NDD classifier were effective in differentiating LBD from the other cohorts. While fairly robust consistencies were observed longitudinally in both the LBD and AD test-retest profiles, cross-validation studies are needed to demonstrate the reported accuracy was not a result of overfitting. Reliance on the MMSE instead of more detailed neuropsychological testing to screen the CG was also a limitation. Several CG participants included in the model development data set were likely misclassified at baseline, as several CG participants were assigned classifications suggestive of covert underlying disease. Longitudinal follow up and additional detailed clinical assessments are necessary to clarify whether any of the CG participants develop NDD.

In this study, we illustrated the potential utility of profiling NDD based on sleep biomarkers. Future studies that include more detailed assessments and confirmation of NDD diagnosis using imaging and biofluid biomarkers and longitudinal sleep assessments will be necessary to further validate the proposed NDD classifier.

## Data Availability

The data underlying this article will be shared on reasonable request to the corresponding author.
